# Development of an eHealth Readiness Assessment Framework for Botswana and Other Developing Countries: Interview Study

**DOI:** 10.2196/12949

**Published:** 2019-08-22

**Authors:** Kabelo Leonard Mauco, Richard Ernest Scott, Maurice Mars

**Affiliations:** 1 Department of TeleHealth University of KwaZulu-Natal Durban South Africa; 2 Department of Health Information Management Botho University Gaborone Botswana; 3 NT Consulting - Global e-Health Inc Calgary, AB Canada; 4 Department of Community Health Sciences University of Calgary Calgary, AB Canada

**Keywords:** eHealth, eHealth readiness, frameworks, Botswana, developing countries

## Abstract

**Background:**

Electronic health (eHealth) readiness has been defined as the preparedness of health care institutions or communities for the anticipated change brought about by programs related to information and communication technology use. To ascertain the degree of such preparedness, an eHealth readiness assessment (eHRA) is needed. Literature on the existing eHRA frameworks and tools shows high inconsistency in content, definitions, and recommendations, and none have been found to be entirely suitable for assessing eHealth readiness in the context of developing countries. To develop an informed eHRA framework and tools with applicability to Botswana and similar developing countries, insight was sought from a broad spectrum of eHealth key informants in Botswana to identify and inform relevant issues, including those not specifically addressed in available eHRA tools.

**Objective:**

The aim of this study was to evaluate key informant (local expert) opinions on aspects that need to be considered when developing an eHRA framework suitable for use in developing countries.

**Methods:**

Interviews with 18 purposively selected key informants were recorded and transcribed. Thematic analysis of transcripts involved the use of an iterative approach and NVivo 11 software. The major themes, as well as subthemes, emerging from the thematic analysis were then discussed and agreed upon by the authors through consensus.

**Results:**

Analysis of interviews identified four eHealth readiness themes (governance, stakeholder issues, resources, and access), with 33 subthemes and 9 sub-subthemes. A major finding was that these results did not directly correspond in content or order to those previously identified in the literature. The results highlighted the need to perform exploratory research before developing an eHRA to ensure that those topics of relevance and importance to the local setting are first identified and then explored in any subsequent eHRA using a locally relevant framework and stakeholder-specific tools. In addition, seven sectors in Botswana were found to play a role in ensuring successful implementation of eHealth projects and might be targets for assessment.

**Conclusions:**

Insight obtained from this study will be used to inform the development of an evidence-based eHealth readiness assessment framework suitable for use in developing countries such as Botswana.

## Introduction

Electronic health (eHealth) readiness has been defined as the preparedness of health care institutions or communities for the anticipated change brought by programs related to Information and Communication Technology (ICT) use [1]. In order to ascertain the degree of such preparedness, an eHealth readiness assessment (eHRA) is needed. The advantages of conducting an assessment of electronic readiness include avoiding substantial loss of time, money, and effort; avoiding delays and disappointment among planners, staff, and users of services; and facilitating the process of change in institutions and communities from contemplation to preparation for ICT implementation [2]. As such, it is critical for an eHRA to be undertaken prior to implementation of any eHealth innovation. The literature has consistently shown consensus regarding the need for proper and holistic eHRA [1,3,4].

In developing countries, eHealth is largely funded by external donors and governments, which is different from the case in developed countries; the health concerns and needs are also different [5]. This therefore requires an approach to eHRA that takes this difference into account. This paper uses Botswana as a case study and develops an approach to eHRA that considers this perspective.

Reliability of the findings of an eHRA are only as good as the framework and tools deployed. Identifying the right framework and tools is a complex process, as there are several eHRA frameworks and associated tools presented in the literature, [6] and no standard framework or tool has yet been described. A recent review analyzed published eHRA frameworks and found none to be entirely suitable to assess eHealth readiness in the context of developing countries [6]. Another review presented a rank order of seven readiness themes according to prevalence in the literature: technological readiness, core/need/motivational readiness, acceptance and use readiness, organizational readiness, information technology skills/training/learning readiness, engagement readiness, and societal readiness [7]. eHealth readiness has extended as far as considering environmental issues [8]. It can be concluded from this and other literature that existing eHealth readiness assessment frameworks and tools show great inconsistency in content, definitions, and recommendations. The literature also demonstrates a need for the readiness frameworks and tools used, and readiness aspects applied, to be context-specific for the setting being considered and the stakeholder groups involved [6].

Botswana, like many developing countries, has also recognized the need for eHealth implementation [9]. Botswana has yet to undertake an eHealth readiness assessment prior to implementation of its eHealth services. Unfortunately, there is no comprehensive eHealth readiness assessment framework suitable for use in developing countries [6]. To develop an informed eHealth readiness assessment framework applicable to Botswana and informative to similar developing countries, insight was sought from a broad spectrum of eHealth key informants (local experts) in Botswana to identify and inform any issues not specifically addressed in available eHRA frameworks.

The aim of this study was to critically analyze eHealth readiness themes emerging from interviews with various eHealth key informants in Botswana and to assesses their relevance to contributing toward the development of a comprehensive and evidence-based eHRA framework for use in Botswana.

## Methods

Interviews were conducted with purposively selected key informants—individuals and organizations perceived to have a role in the implementation of eHealth in Botswana. Prospective key informants were contacted in-person, by email, or by telephone and, after explanation of the study, invited to provide consent and participate in the study. Key informants (local experts) interviewed were a director from the Botswana communications regulatory authority, three heads of district health management teams, three hospital managers, three hospital ICT managers, three community leaders, and five people with relevant experience in electronic solutions (e-solutions). The latter included a former director of e-solutions for a large national bank, the head of planning technology for a telco, the head of telemedicine and informatics for an academic partnership, an informatics unit director, and an ICT coordinator for a relevant ministry. A total of 18 interviews were conducted with some key informants based in rural settings (n=8) and others in urban settings (n=10) across Botswana.

Face-to-face structured interviews using an interview guide with open**-**ended questions were performed at locations convenient to the participant. The interview questions were developed based on the aim of the study and interview tools identified during the literature review [1,10,11]. Interviews were recorded and transcribed. Where interviewees responded in Setswana (the local language), back translation was completed, with discrepancies in responses settled through mutual consensus between the translators involved. Thematic analysis of transcripts involved the use of an iterative approach and NVivo software [computer program] (Version 11. Melbourne, Australia: QSR International Pty Ltd; 2015). The four major themes, as well as subthemes, emerging from the thematic analysis were then discussed and agreed upon by the authors through consensus.

Ethical approval for the study was obtained from both the Botswana Ministry of Health and the University of KwaZulu-Natal. All participants provided written informed consent before participating in the study.

## Results

Analysis of interviews identified four eHealth readiness themes of governance, stakeholder issues, resources, and access, each with several subthemes (Textbox 1).

**Electronic health readiness themes and subthemes from expert interviews.**

**Governance**:

- National governance
  - Political will
  - Legal framework
  - Implementation plan
  - Public private partnerships
  - e-Governance
    - eHealth leverage
  - Health care service delivery
    - Unique patient identifier
    - Population distribution
    - Health facility distribution
  - Power supply

- Institutional governance
  - Policies
  - Regulations
  - Interoperability
  - Data stewardship
  - Security for eHealth resources

**Stakeholder Issues:**

- Engagement
- Public awareness
- Readiness
- Change management

**Resources:**

- Budget
- Information and communication technology infrastructure
- Information and communication technology infostructure
  - Electronic health records

- Human resources
  - Human health resources
  - Human eHealth resources

**Access:**

- Literacy
  - Technical literacy

- Training
  - Curriculum

- Network reach
- Internet availability
- Affordability of access to e-media
- Ubiquity of access to e-services
- Access to e-devices
- Presence to access electronic health records
- Availability of eHealth resources in local languages
- Rate of social media usage
- eHealth support

Governance captured various subthemes that the key informants believed needed consideration at both national and institutional levels to ensure eHealth readiness. Stakeholder issues encapsulated subthemes concerned with ensuring that community members were involved during implementation of eHealth projects. Resources identified human, structural, and budgetary subthemes. Access comprised several subthemes concerned with ensuring all community members (eg, citizens and health care workers) were able to access eHealth services.

The key informants considered seven sectors in Botswana to play a role in ensuring successful implementation of eHealth projects (Textbox 2).

**Figure 1:**
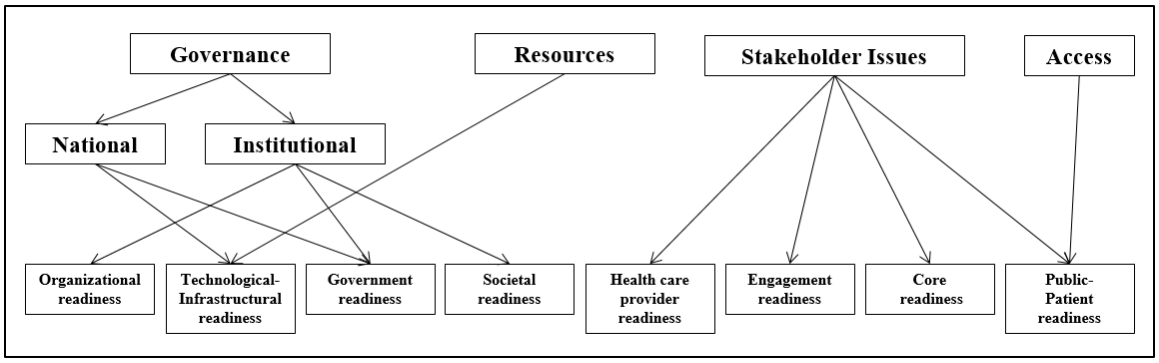
The four eHealth readiness themes identified from expert interviews (above) mapped to eHealth readiness types identified from the literature (below).

These were communities, government, private sector, state-owned enterprises, statutory corporations, international agencies, and international partnerships. The eHealth readiness assessment types derived from the literature [6] and eHealth readiness themes obtained from key informant (expert) interviews were compared and mapped with each other (Figure 1).

Figure 1 compared and mapped eHealth readiness themes identified from expert interviews and eHealth readiness types identified from the literature. To provide uniformity, specific definitions [6] were applied to each eHealth readiness type as follows:

- Organizational readiness: Gauges the extent to which the institutional setting and culture supports and promotes awareness, implementation, and use of eHealth innovations (eg, presence of relevant policies and senior management support).
- Technological-infrastructural readiness: Gauges the availability and affordability of ICT resources necessary to implement a proposed eHealth innovation (eg, skilled human resources, ICT support, quality ICT infrastructure, and power supply).
- Government readiness: Gauges the extent to which a country’s government and politicians support and promote awareness, implementation, and use of eHealth innovations (eg, presence of relevant policies and funding).
- Societal readiness: Gauges the degree of “interaction” associated with a health care institution. Interaction is described by three parameters: interaction among members of a health care institution, interaction of a health care institution with other health care institutions, and interaction of a health care institution with its local communities.
- Health care provider readiness: Gauges the influence of a health care provider’s personal experience, primarily their perception and receptiveness toward the use of eHealth technology.
- Engagement readiness: Gauges the extent to which members of a community are exposed to the concept of eHealth and are actively debating its perceived benefits as well as negative impacts. It also involves gauging the willingness of members of a community to accept training on eHealth.
- Core readiness: Gauges the extent to which members of a community are dissatisfied with the current status of their health care service provision, see eHealth as a solution, and express their need and preparedness for eHealth services.
- Public-patient readiness: Gauges the extent to which members of the public and patients are aware of, and can afford and access, eHealth services. It also involves gauging the influence of their personal experiences on their perception and receptiveness toward the use of eHealth technology.

**Key informants’ opinion on principal persons/organizations that need to be considered for successful implementation of eHealth in Botswana.**

**National sectors:**

- Communities
  - Chiefs
  - Councilors
  - Community members

- Government
  - Ministry of health
  - Communications ministry
  - Infrastructure ministry
  - Ministry of finance
  - Ministry of education
  - Ministry of agriculture
  - Parliament
  - Media
  - Libraries
  - Schools

- Private sector
  - Private health care providers
  - Mobile network operators
  - Telco industry
  - Technology developers
  - Financial industry
  - Medical aid providers
  - Media
  - Libraries
  - Schools

- State-owned enterprises
  - Telco provider
  - Electric utility
  - Postal service provider

- Statutory corporations
  - Communications regulatory authority

**International sectors:**

- Agencies
  - International Telecommunications Union
  - World Health Organization

- Partnerships
  - Centre for Disease Control and Prevention
  - Botswana-UPenn Partnership
  - Botswana-USA Partnership

## Discussion

This study identified four eHealth readiness themes (governance, stakeholder issues, resources, and access), 33 subthemes (including national and institutional governance), and 9 sub-subthemes of relevance and importance to Botswana (Textbox 1). A major finding was that these results did not directly correspond in content or order to those previously identified in recent literature reviews [6,7]. This highlights the need to perform exploratory research before developing an eHRA to ensure that those topics of relevance and importance to the local setting are first identified and then explored in any subsequent eHRA. Once areas of poor readiness have been identified, actions that lead to improved readiness can be implemented.

To encourage greater consistency in use of terms and application of eHRA frameworks, clear definitions of several types of eHealth readiness, derived from the literature [6], were aligned and mapped with the eHealth readiness themes identified from the key informant interviews (Figure 1).

The theme of governance that emerged from the interviews was split into two major subthemes (national governance and institutional governance) to cater to responses related to issues of governance in a country and the health care institution level, respectively. These subthemes corresponded to elements within the definitions from the literature of “organizational readiness,” “technological/infrastructural readiness,” “societal readiness” and “government readiness” types (Figure 1) [6]. Governance has been defined as the exercise of political and administrative authority at all levels to manage a country’s affairs [12]. Whether at the national or institutional level, entities involved with eHealth implementation need to positively enforce their political and administrative authority in order to manage and ensure successful implementation of eHealth. To a large extent, and considering the dominant role of government and donors in developing countries, this will be dependent on how much these stakeholders are involved and prepared to assist in this regard. Hence, with regard to a country, there will be a need for the government itself to display readiness. In addition, at both national and institutional levels, the entities concerned need to have measures in place on how they will ensure interaction among all the parties necessary for a successful eHealth implementation (ie, societal readiness). As a result, the theme of “governance” encapsulated the four literature eHealth readiness types described (organizational readiness, technological/infrastructural readiness, societal readiness, and government readiness) [6].

Kierkegaard [13] affirms the role of governance in eHealth readiness by stating that the dynamic relationship between governance and eHealth plays a critical role in terms of implementation success and failure. The subtheme of national governance and its associated components captures the literature definitions of “government readiness” as well as “technological/infrastructural readiness” (Figure 1). Although government readiness was not a commonly cited eHealth readiness type among the eHealth readiness assessment frameworks previously reviewed [6], some of the key informants emphasized the importance of this eHealth readiness type toward the success of eHealth implementation. This might be relevant in developing countries such as Botswana where the government is often the major custodian of health care services [14]. Indeed, one respondent noted that the “public healthcare sector is huge and it serves majority of the population.”

Given the bureaucratic and intertwined nature of any government, with multiple government ministries and departments, government readiness must also involve preparedness of these branches for the successful implementation of eHealth. The government organs highlighted by the key informants interviewed are Ministry of Health (typically the custodian of eHealth projects); communications and infrastructure ministries (Ministry of Infrastructure, Science and Technology, and Ministry of Transport and Communications), which provide a platform for the support of the eHealth technology; the Ministry of Education and Skills Development that ensures that issues of technical literacy are addressed so that end users are eHealth ready; and the Ministry of Agriculture (sometimes the custodian of projects involving animal and plant eHealth) (Textbox 2). Lastly, one of the crucial Ministries required to be involved to ensure government readiness as a financier for any eHealth project is the Ministry of Finance and Development Planning. Any successful eHealth implementation approach requires a synergetic partnership between all parties in the government [13].

The only component of national governance not explicitly addressed by the definitions of “government readiness” and “technological readiness” is the issue of public private partnerships (PPP), which was raised by some of the key informants. Possible partners within any PPP could include private health care providers, mobile network operators, telcos, technology developers, financial entities, and medical aid providers (Textbox 2). In developing countries where resources are limited, successful implementation of eHealth may greatly benefit from PPP. The importance of such partnerships was emphasized by their inclusion within the draft eHealth strategy document for Zimbabwe [15].

Notably absent in the interviews and the eHealth readiness literature review was the role of eHealth strategy as a driver of successful eHealth implementation. Presence of an eHealth strategy serves to guide eHealth implementation and inform setting up a relevant regulatory/legal framework in a country [16]. The importance of a national eHealth strategy in strengthening eHealth implementation is also emphasized in the national eHealth strategy toolkit of the World Health Organization (WHO) and International Telecommunication Union (ITU) [17].

The subtheme of institutional governance, and its associated components, captured the definitions of organizational readiness and societal readiness from the literature (Figure 1). However, both definitions of organizational readiness and societal readiness lacked explicit mention of interoperability as a means of attaining eHealth readiness. The issue of interoperability emerged during interviews under the subtheme of institutional governance. One key informant stated, “We currently have so many systems in place, we need to find out if they are able to speak to each other and if there is a backup system.” Interoperability has been defined as the extent to which systems and devices can exchange data and interpret the shared data [18]. Most developing countries including Botswana are, or have been, recipients of eHealth systems from foreign donors and international partnerships. This results in the presence of a number of systems that are unable to communicate with each other. Hence, interoperability is an issue that needs to be addressed in any eHealth readiness assessment framework meant for developing countries. The importance of interoperability can be estimated by the fact that it is specifically mentioned in the WHO and ITU National eHealth Strategy Toolkit as one of the eHealth components to be addressed in the development of a national eHealth vision [17].

The theme of stakeholder issues, and its subthemes emerging from the interviews, corresponded with the eHealth readiness types from the literature of health care provider readiness, engagement readiness, core readiness, and public patient readiness (Figure 1) [6]. These associated types of eHealth readiness only recognize members of the public and health care workers as stakeholders who need to be prepared for the implementation of eHealth. However, other stakeholders of relevance emerged from the interviews, such as the private sector, state-owned enterprises, statutory corporations, international agencies, and international partnerships. The need for a holistic approach has also been emphasized previously [16,17,19].

All relevant stakeholders need to be engaged from the inception of a national eHealth strategy to ensure that their interests are understood and addressed, including the benefits that may be delivered to each stakeholder group. They must also remain informed on progress to ensure the vision (eHealth implementation) has their continued support, and each group remains involved in the planning and delivery of the vision itself [17]. In 2005, the World Health Assembly called upon member nations to create national centers or networks of excellence for eHealth [20]. Kwankam [21] has also proposed a need for a well-organized framework for a national infostructure for eHealth, comprising a national eHealth council (government advisors), an eHealth corps (body of professional eHealth workers), eHealth steering committee (national and regional Ministry of Health advisors), and an eHealth center/network of excellence (to foster eHealth research and best practice). In addition, Kwankam recommended creation of a national eHealth society to act as a forum in each country for eHealth professionals to exchange ideas and share knowledge [21].

An issue previously noted is the process by which stakeholder engagement is carried out, especially in many developing countries, where social structures will play a role in successful stakeholder engagement [6]. Some key informants addressed this issue of sociocultural readiness by noting, “One must follow the cultural protocol of consulting that is, through the chiefs or village leaders.” Another stated that “In our culture, any new development introduced into a community must first be with consent from the community leader.” Such considerations are a concern for eHealth readiness that is not typically given sufficient priority, although Khoja et al [1] considered it a part of societal readiness. Successful implementation of eHealth involves readiness by a number of stakeholders. As previously discussed, assessing the readiness of such a variety of stakeholders must involve the use of separate eHealth readiness assessment tools for the appropriate groups to complete [6]. This needs to be done, for example, to avoid a situation where eHealth readiness assessment tools for technical individuals are similar to those for managers or policy makers.

The theme of resources only corresponded to the definition of technological/infrastructural readiness (Figure 1). Technological/infrastructural readiness was defined in the literature as gauging the availability and affordability of ICT resources necessary to implement a proposed eHealth innovation [6]. The definition seems to be more concerned with ICT resources and does not adequately address the need for other resources such as a budget specific for eHealth, ICT infostructure, and the relevant human resources. A specific budget for eHealth is crucial for sustainability of a project and must be determined as part of the business plan prior to embarking on eHealth implementation. Equally important is the availability of sufficient and appropriate human health resources, or more specifically, human eHealth resources (ie, professionals knowledgeable and trained in eHealth). Infostructure is an ill-defined term, but has been considered as all needs beyond physical hardware and software infrastructure. Despite its ephemeral nature, it is an important inclusion as a factor determining readiness.

These issues (budget, infostructure, and human eHealth resources) may be of greater concern for developing countries. For example, most sub-Saharan African countries are economically constrained; face a critical shortage of health care workers, in general; and have a disparate burden of disease [22]. Such issues should be highlighted in any eHealth readiness assessment framework for developing countries, as they could negatively affect eHealth implementation. Notably, the type of resources required to enable successful implementation of eHealth ultimately depends on the type of eHealth solution to be deployed.

The theme of access and its subthemes corresponded best to the definition of public-patient readiness (Figure 1), even though the definition of public-patient readiness does not adequately capture some of the components highlighted under the theme and subthemes of access. In most communities in developing countries, especially in the rural areas, local access to ICT equipment and facilities is a challenge [23]. In rural Botswana, it is not uncommon for the only place to have internet connectivity to be government institutions such as public schools, public hospitals, libraries, and post offices. This constrains access to any eHealth services by end users and negatively impacts the success of eHealth implementations in developing countries. Therefore, in the context of developing countries, eHealth readiness might be gauged by the availability of public places where internet services could be accessed for free.

Less traditional parameters have yet to be considered as indicators of readiness, particularly for developing countries. A recent systematic review described how mobile health (mHealth) has evolved over the years in terms of mobile devices employed [24]. The research illustrated how mHealth interventions have progressed from requiring the use of basic phones and feature phones to smart devices. One respondent noted that continuity of such a trend may actually negatively impact eHealth implementation in developing countries: “Mobile devices are also expensive here (Botswana) as compared to other countries such as South Africa and east African countries.” This is because of several issues, including the need to import devices, the lack of attention to developing market needs, the ongoing trend of mHealth being smartphone dependent, and the inability of the populace to afford such devices. In addition, as technology requirements become more sophisticated and complex, the devices become more expensive, making them even less affordable to most people in developing countries. This makes affordability and access to devices a potential measure of eHealth readiness for developing countries.

Literacy has been identified as an issue in developing countries [25]. This study also identified lack of literacy as an issue, with a participant noting, “Another challenge is that of education level. If you go to villages you will find a lot of people that are illiterate and not sensitized to the benefits of electronic communications.” Lack of basic literacy, technical literacy, and health literacy, as highlighted during the interviews, can also contribute to denying the populace access to eHealth services. Measures of such types of literacy also need to be incorporated into any eHealth readiness assessment framework and tool. This is associated with the need to ensure that eHealth resources can be accessed in local languages.

The interviews provided insight of what participants thought needed to be considered when assessing eHealth readiness. However, as shown above, additional issues exist and need to be considered when developing an eHealth readiness assessment framework for developing countries such as Botswana.

In conclusion, the importance of and need for eHealth readiness assessment prior to eHealth implementation attempts are well established [26]. This study has confirmed that a plethora of issues influence the readiness of a setting and that issues of most relevance locally must be those assessed in any given situation. Furthermore, the study re-enforces the need to identify different stakeholder groups and then assess issues relevant to each group by using group-specific assessment tools. The process adopted for this study has established a unique and locally informed evidence base for issues not recognized in current eHRA frameworks. This process should be replicated elsewhere in developing countries. As a consequence, insight from this study can be used to support successful eHealth implementation by development of evidence-based eHealth readiness assessment framework specific to Botswana or other developing countries and settings.

## Abbreviations

CDC: Centre for Disease Control
eHealth: electronic health
eHRA: e-Health Readiness Assessment
ICT: Information and Communication Technology
ITU: International Telecommunications Union
mHealth: mobile health
PPP: Public Private Partnerships
WHO: World Health Organization
